# Exploring the Incidence and Associated Risk Factors of Barrett’s Esophagus in African Americans: A Retrospective Study

**DOI:** 10.29011/2690-9480.100164

**Published:** 2023-08-03

**Authors:** Hassan Ashktorab, Sahar Geramfard, Mudasir Rashid, Rumaisa Rashid, Swetha M Mynedi, Mehdi Nouraie, Ali Nezamloo, Hassan Brim

**Affiliations:** 1 Department of Medicine and Cancer Center, Department of Pathology, Howard University College of Medicine, Washington DC, USA; 2 Division of Pulmonary, Allergy and Critical Care Medicine, Department of Medicine University of Pittsburgh, PA, USA

**Keywords:** Barrett, African American, Esophageal Gastro Endoscopy (EGD), Esophageal Adenocarcinoma (EAC) and Squamous Cell Carcinoma (SCC), *Helicobacter pylori*

## Abstract

**Background:**

The prevalence of Barrett’s esophagus (BE) in African Americans (AA) is uncertain. However, several potential risk factors, includes family history, male sex, ethnicity, chronic heartburn and acid reflux, age over 60, current or past smoking, and obesity are associated with BE. The aim of this study is to determine the incidence of BE in AA patients who underwent Esophageal Gastro Endoscopy (EGD).

**Methods:**

Medical and demographic data of 1,253 AA patients with various symptoms, including BE, Esophageal adenocarcinoma (EAC), esophageal squamous adenocarcinoma (SCC), hiatal hernia, *H. pylori* infection, Gastro-Esophageal Reflux Disease (GERD), Gastritis, and esophagitis, were collected from January 2004 to December 2014 at Howard University Hospital.

**Results:**

Among the 1,253 patients, the median age was 61 and 49% were male out of the total. The frequencies of EAC (p= 0.05), and SCC (p= 0.002) were significantly high in males, along with SCC frequency significantly increased with older age (p<0.001). Furthermore, esophageal polyps with hiatal hernia (p=0.008) and *H. pylori* (p=<0.001) were found to be associated with esophagitis, and its presence may contribute to the development of BE.

**Conclusion:**

The findings highlighted the high prevalence of GERD symptoms and pathologic gastritis along with EAC was less common than SCC and both types of esophageal cancer were associated with male gender and older age whereas, H. pylori infection was identified as a risk factor for pathologic gastritis in AA. Overall data emphasize the need for extensive research, increased awareness, diagnosis, and management of GERD, gastritis, and related conditions to uncover the underlying mechanisms and factors contributing to these conditions in the AA population.

## Introduction

There is a spectrum from Non-Erosive Reflux Disease (NERD), Gastro-Esophageal Reflux Disease (GERD), Erosive Esophagitis (EE), Ulcerative Esophagitis (UE), Esophageal Stricture (ES), Barrett’s Esophagus (BE) to Esophageal Adenocarcinoma (EAC) [1–6]. However, not all patients with GERD and EE develop BE, and not all patients with BE have a history of GERD [7–9]. BE is defined as metaplastic changes from squamous epithelium to columnar epithelium in the distal esophagus [9]. Transformation from squamous epithelium to columnar accompanied with goblet cells, diagnosed by biopsy, is an adaptive mechanism [10]. When visible upon, this term ‘‘Erosive esophagitis’’ is the visible mucosal damage in upper Gastrointestinal endoscopy. An association between BE and older age is well established [11–13]. It is also believed that BE is mostly a White Caucasian males’ disease This may reflect genomic variants associated with European descendancy [9, 14]. A study has shown that more than 50% of prevalent BE in population-based communities is undiagnosed, hence, reducing the proportion of BE under surveillance [15, 16]. Male sex, age over 50, smoking, White race, chronic reflux symptoms, central obesity, and family history are associated with BE [17–21]. It has been reported that the prevalence of BE in those without GERD symptoms was low (0.8%), however, a higher prevalence in those with known risk factors is observed in people with age over 50 years (6.1%), male sex (6.8%), obesity (1.9%), family history of BE/EAC (23%), and GERD (2.3%) [18, 19, 22, 23].

BE is diagnosed by Esophagogastroduodenoscopy (EGD) in patients with upper Gastrointestinal (GI) symptoms and during screening or surveillance [24, 25]. There are many factors that contribute to the natural history of BE and EAC including age, race, visceral obesity, Waist-Hip Ratio (WHR), food preferences, esophageal acid clearance, delayed gastric emptying, hiatal hernia, and diversely infection with *H. pylori*. The prevalence of BE and EAC is increasing in the USA, Western Europe, and Australia [26–29]. Even though they are increasing in African Americans, the rate of increase in prevalence is lower than Non-Hispanic Whites (NHW).

Additionally, numerous studies have shown that different ethnic groups have different prevalence rates for Barrett’s esophagus. For instance, a Taiwanese group reports prevalence rates for erosive esophagitis and Barrett’s esophagus of up to 15% and 2%, respectively. A Malaysian study shows a prevalence rate of 6% for endoscopically documented esophagitis. In addition, Ho et al. found that Indians had a 1.6% prevalence of reflux symptoms, which was twice the incidence of Chinese. Additionally, compared to the West, Singapore’s general population had a lower prevalence of all chronic gastrointestinal symptoms; for example, the incidence of endoscopic reflux esophagitis among Chinese people is 5%, which is lower than in Western nations, suggesting that racial differences in Barrett’s esophagus exist [23, 30–35]. We have conducted a study in African Americans to investigate the relation of race/ethnicity and other factors to BE during 11 years of EGD data at our hospital.

## Methods:

### Patients:

We collected and reviewed electronic health and pathological records from 1,253 African Americans at Howard University Hospital, Washington DC from January 2004 to December 2014. The study was approved by Howard University Institutional board review (12-MED-79). All 1253 patients, with or without upper gastrointestinal symptoms, underwent EGD. Data were extracted from the Howard University Hospital electronic health record system. The following data were recorded: age, gender, clinical presentations, GERD symptoms, pre and post EGD diagnosis and endoscopic findings including Barrett’s Esophagus (BE), Esophageal Adenocarcinoma (EAC), Squamous Cell Carcinoma (SCC), gastritis, hiatal hernia, *H. pylori* infection. A subject was considered to have definitive BE if intestinal columnar epithelia including goblet cells is confirmed by pathology based on earlier reports [10].

### Ethical Statement:

The authors are accountable for all aspects of the work in ensuring that questions related to the accuracy or integrity of any part of the work are appropriately investigated and resolved. This study was conducted in accordance with the Declaration of Helsinki (as revised in 2013) and was approved by the Ethics Committee of Juntendo University Faculty of Medicine (# 17–004). Informed consent was obtained from all patients.

### Statistical Analysis:

Continues variables were presented by median (interquartile range). Categorical variables were tested between groups of patients using Chi-2 or Fisher’s exact test.

## Results

### GERD and gastritis but not BE was among the top clinical features

Previous research has indicated that GERD and gastritis are common gastrointestinal conditions that can have similar symptoms [36, 37]. However, these conditions have distinct causes, treatments, and can vary among different populations, including different ethnic and racial groups. In our study, we examined a large cohort of African American individuals, consisting of 1,253 participants, with 610 (49%) being male and 641(51%) with a median age of 61 years (InterQuartile Range: 52–70). Among these individuals, 516 (41.1%) presented with GERD symptoms such as heartburn, chest pain, and dysphagia, while 737 (58.9%) did not experience any symptoms. In our cohort, 278 individuals (22%) were diagnosed with hiatal hernia, and 224 individuals (23%) tested positive for H. pylori infection based on gastric biopsy stain results (Table 1). Endoscopic examination (EGD) revealed that 430 individuals (34%) had esophageal tissue inflammation, and 874 individuals (63%) had gastric tissue inflammation. The pathology findings indicated that 8 individuals (0.6%) had Barrett’s esophagus (BE), 13 individuals (1.0%) had esophageal adenocarcinoma (EAC), 36 individuals (2.9%) had squamous cell carcinoma (SCC) of the esophagus, and 448 individuals (42%) had pathologic gastritis (Table 1).

These findings provide valuable insights into the demographic, clinical, and pathological characteristics of GERD, gastritis, and related conditions in the African American population. Overall, these findings are significant as they shed light on the prevalence and risk factors of gastrointestinal conditions in the AA population, emphasizing the importance of further research, diagnosis, and management of GERD-related disorders and related complications in this specific ethnic group and varies in other groups.

### Hiatal hernia is more prevalent in esophagitis patients:

**Figure 1:**
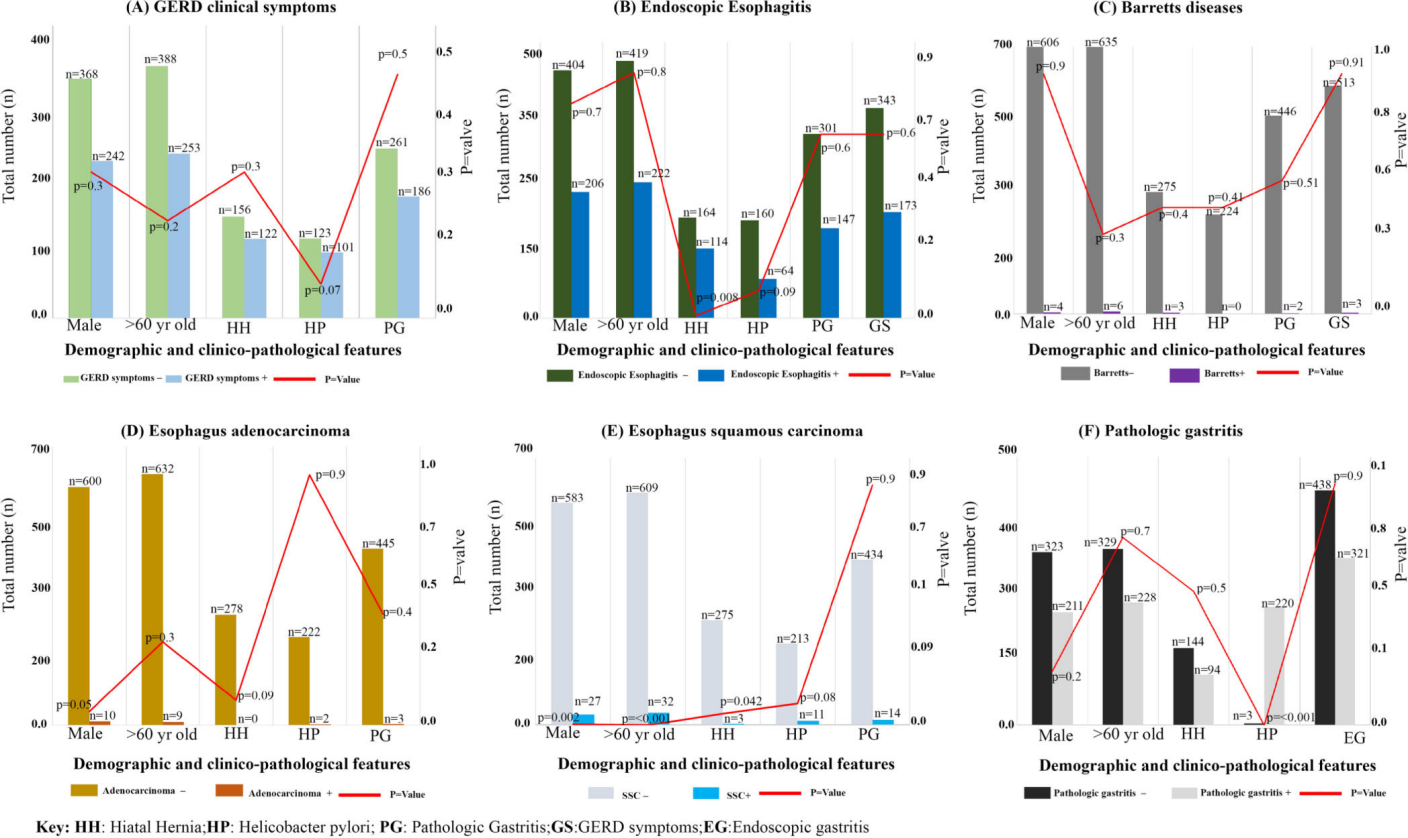
Demographic and clinico-pathological parameters of AA individuals: **(A)** Bar graph depicts negative (light green color) or positive (light blue) for GERD clinical symptoms of individuals respectively; (B) Bar graph shows absence (Dark green) or presence (dark blue color) of Endoscopic Esophagitis in individuals, respectively; (C) Bar graph demonstrates absence (grey color) or presence (violet color) of Barrett’s disease in individuals respectively; (D) Bar graph depicts absence (−) presence (+) Esophagus adenocarcinoma individuals; (E) Bar graph depicts absence (−) presence (+) Esophagus squamous carcinoma individuals; (F) Bar graph depicts absence (−) presence (+) Pathologic gastritis individuals; The Y-axis represents the total number of individuals(n=1253) and P value (red line); X-axis represents the Demographic and clinico-pathological parameters including Male, age, Hiatal hernia, Helicobacter pylori and pathologic gastritis and GERD symptoms.

In the study, a total of 516 patients with GERD symptoms, 242 (47%) were male, 253 (49%) were over 60 years old, 122 (24%) had hiatal hernia, 101 (26%) were infected with *H. pylori* and 187 (43%) had pathological gastritis (Figure 1A; Supplementary Table 1A). From 737 patients, without GERD symptoms, 368 (50%) were male, 388 (53%) were over 60 yrs, 156 (21%) had hiatal hernia and 123 (21%) displayed *H. pylori* infection, and 261 (41%) had gastritis on collected gastric biopsies. These findings indicate a higher prevalence of hiatal hernia in individuals with esophagitis, regardless of the presence of GERD symptoms. It is important to note that the study did not establish significant associations between gender, age, gastritis, H. pylori infection, and either symptomatic or endoscopic esophagitis. However, it is essential to consider that other factors may still contribute to the development of esophagitis, even though they were not identified as significant in this study. Based on the study’s observations, it was noted that hiatal hernia appears to be more prevalent in African American individuals with esophagitis. This suggests a potential association between hiatal hernia and esophagitis in this population. Further research and investigation are needed to gain a better understanding of the relationship between hiatal hernia and esophagitis in African American individuals, as well as to explore any underlying factors that may contribute to this association.

Furthermore, Endoscopic examination data were analyzed, among the 430 patients diagnosed with esophagitis through esophagogastroduodenoscopy (EGD), 206 (48%) were male, and 222 (52%) were over the age of 60. Out of these patients, 114 (27%) had hiatal hernia, 64 (19%) was found to be infected with H. pylori,147 (40%) had pathological gastritis and 173 (40%) showed GERD symptoms . There was no significant association found between H. pylori infection and esophagitis (p=0.09). However, a significant association was observed between hiatal hernia and esophagitis (p=0.008). In the group of 823 patients without EGD-diagnosed esophagitis, 404 (49%) were male with 419 (51%) over the age of 60 years. Among them, 164 (20%) had hiatal hernia, 160 (24%) were infected with H. pylori, 301 (42%) had pathological gastritis findings, and 343 (42%) presented with GERD symptoms (Figure 1B; Supplementary Table 1B). The study demonstrated that hiatal hernia was more prevalent in patients with endoscopic esophagitis (114 cases, 27%) compared to those without endoscopic esophagitis (164 cases, 20%), indicating a significant association between hiatal hernia and the presence of endoscopic esophagitis (p=0.008).

In our study, the number of patients with confirmed Barrett’s esophagus (BE) was low, with only 8 (0.6%) individuals having pathological confirmation of BE. Among these BE patients, the distribution between genders was equal. Out of the 8 patients with BE, 4 (50%) was male, 6 (75%) were over the age of 60, and 3 (38%) had hiatal hernia. None of the BE patients were found to be infected with H. pylori. Two (25%) of the BE patients also had pathologic gastritis, and only 3 (38%) patients presented with GERD symptoms (Figure 1C; Supplementary Table 1C). These findings support the understanding that BE is more commonly seen in older individuals and may be associated with the presence of hiatal hernia. The absence of H. pylori infection in the BE patients suggests that other factors, such as chronic acid reflux, may play a significant role in the development of BE. Additionally, the coexistence of pathologic gastritis in some BE patients highlights the complex nature of these gastrointestinal conditions.

In addition to the factors previously mentioned, other clinical and pathological parameters were also considered and associated in this study. One notable finding was a higher prevalence of squamous cell carcinoma (SCC) compared to esophageal adenocarcinoma (EAC) in African Americans. thirteen patients (1%) were diagnosed with EAC based on pathology biopsies, indicating a low prevalence of EAC in this population, which is consistent with previous reports [38, 39]. Among the EAC patients, 10 (77%) were males, and 9 (69%) were over the age of 60. None of the EAC patients had hiatal hernia, 2 (22%) had H. pylori infection, and 3 (27%) had gastritis. On the other hand, 36 patients were diagnosed with SCC according to (Figure 1D and E and Supplementary Table1D). Among these SCC patients, 27 (75%) were male (p=0.002), and 32 (89%) were over the age of 60 (p<0.001). Three (8%) of the SCC patients had hiatal hernia, 11 (35%) were infected with H. pylori (p=0.08), and 14 (42%) had gastritis(Table 4). Furthermore, the prevalence of H. pylori infection among patients without EAC was 23% (222 cases), and no significant association was found between H. pylori infection and EAC (p=0.9). These findings indicate that SCC was more prevalent than EAC in African American patients. Male gender and older age were associated with both SCC and EAC. The presence of hiatal hernia, H. pylori infection, and gastritis varied among the different types of esophageal cancers but did not show consistent significant associations. It’s important to note that these observations contribute to our understanding of esophageal cancer in the African American population, but further research is needed to fully elucidate the underlying factors and mechanisms contributing to these associations.

Furthermore, *H. pylori* infection was identified as a risk factor for the development of pathologic gastritis, which was found to be more prevalent in African Americans [40]. In this study, out of all the patients included, 448 (42%) were diagnosed with gastritis based on biopsy results. Among these patients with gastritis, 228 (51%) were over the age of 60, 94 (21%) had hiatal hernia, 220 (58%) were infected with H. pylori (p<0.001), and 312 (70%) exhibited inflammation during esophagogastroduodenoscopy (EGD) examination (Figure 1F and Supplementary Table 1D). On the other hand, among the 631 patients who did not show evidence of gastritis on biopsy, 323 (51%) were male, 329 (52%) were over the age of 60, 144 (23%) had hiatal hernia, only 3 (0.5%) were infected with H. pylori, and 438 (69%) displayed stomach inflammation during EGD (Figure 1F and Supplementary Table 1D). These findings suggest that H. pylori infection is indeed a risk factor for the development of pathologic gastritis and that it is more prevalent among African Americans. The presence of hiatal hernia and the presence of stomach inflammation on EGD were also associated with gastritis. The absence of significant H. pylori infection among patients without gastritis further supports the association between H. pylori and gastritis. It is important to note that these observations provide valuable insights into the relationship between H. pylori infection, gastritis, and relevant clinical factors in this study population. However, further research is needed to better understand the underlying mechanisms and potential implications of these associations.

Overall, these findings highlight the complex interplay of various factors in the development of GERD, esophagitis, BE, and esophageal cancer. The study provides valuable insights into the associations between these conditions and clinical and pathological parameters, particularly in the African American population. Also, the study highlights the need for further research and interventions to improve the diagnosis, treatment, and prevention of these gastrointestinal conditions and calling for further research and interventions in this specific ethnic group like African American population.

## Discussion:

In this hospital-based 11 years study of 1,253 patients’ clinical and pathology reports, more female patients sought EGD screening than males, probably reflecting a better health awareness in females and more resilience in male AA patients as it relates to upper GI discomfort. Additionally, it was found that BE was more likely in males than in females based on histologic evaluation (2.9% vs. 0.8%, P = 0.0001) [41]. Patients undergoing EGD without any symptoms represented more than half of the study cohort (59%). Hiatal hernia, infection with *H. pylori*, endoscopic and pathologic gastritis were found in these 737 symptom-free patients which likely reflect that in the general AA population, many of these findings are underestimated.

In our study, the prevalence of endoscopic 874 (63%), pathologic 448 (42%), gastritis, hiatal hernia 278 (22%) and infection with *H. pylori* 224 (23%) were high, while BE 8 (0.6%), EAC 13 (1.0%) and SCC 36 (2.9%) prevalence were low compared to Non-Hispanic Whites [13]. Patients with GERD symptoms (516) showed more infection with *H. pylori* 101(26%) and pathological gastritis 187 (43%) when compared to NHWs. Although 737 patients had no GERD associated, there were significant rates of *H. pylori* infection 156 (21%) and gastritis on biopsy 261(41%). The prevalence of BE in African Americans has been addressed only in 2 prior published studies [13,42]. In a cross-sectional EGD study conducted at the Michael E. DeBakey Veteran Affairs Medical Center (MEDVAMC) in Houston, Texas, (301 BE cases and 1,651 controls). NHWs displayed a significantly higher BE prevalence than AAs (21.3 vs. 5.0%; P <0.001). Jones et al., 2021 have reported that based on histologically confirmed samples, blacks (n=3,957) had shorter BE lengths (1.61 vs 2.35 cm, P < 0.01) and were less likely with dysplasia than whites (n=96,891) [43].

In our study, there were 8 BE cases in 1,253 patients. From these 8 BE cases, the gender distribution was equal but none of them had infection with *H. pylori*. Ashktorab et al. previously reported that in AAs, *H. pylori* infection was found to be a protective factor for reflux esophagitis which is a precursor for BE. In concordance with our previous report, other recent report demonstrated that the protective factor of BE was differential expression of glutathione S-transferase theta 2 (GSTT2) gene in African Americans compared with European Americans [14].

More than half of cases with BE were older than 60 and 3 (38%) had hiatal hernia. Age over 50 years and hiatal hernia are risk factors for BE both in NHWs and AAs. NHWs were more likely to be male, and less likely to have *H. pylori* infection (P <0.001) [42]. NHWs were more prone to have long-segment BE and dysplasia than AAs. Independent BE risk factors for AAs, hiatus hernia ≥3 cm (OR 4.12; 95% CI, 1.57–10.81) and H. pylori (OR, 0.64; 95% CI, 0.41–0.99) were statistically significantly associated with BE risk for NHWs. Among all cases and controls, race was a risk factor for BE, independent of other BE risk factors (OR for AAs, 0.26; 95%).

A retrospective analysis of 2,100 patients, in Columbia University in New York, who underwent upper endoscopy during a 1-year period consisted of Whites, blacks, and Hispanics. Whites had a significantly higher prevalence of BE than Hispanics (6.1% vs 1.7%, P = .0002) and blacks (6.1% vs 1.6%, P = .004). In multivariable analysis, factors associated with decreased risk of BE were black race (OR, 0.34; 95% CI, 0.12– 0.97) and Hispanic ethnicity (OR, 0.38; 95% CI, 0.18 – 0.84). Male sex (OR, 1.86; 95% CI, 1.20 –2.87), reflux symptoms (OR, 2.87; 95% CI, 1.84 – 4.45), hiatal hernia (OR, 3.53; 95% CI, 2.17–5.72), and older age were associated with increased risk of BE. From 1,245 patients with no BE in our study, 513 (41%) presented with GERD symptoms while only 3 out of 8 (38%) BE patients displayed GERD symptoms. As such, in our cohort, GERD symptoms don’t seem to be associated with BE. In our study, 13 (1%) and 36 (2.9%) patients had EAC and SCC, respectively. The prevalence of EAC was low while that of SCC was high in AA as reported in other studies [44]. Most of the EAC and SCC patients were male and older than 60 years old. From the 1,240 patients without EAC, 445 (42%) had gastritis on biopsy and 222 (23%) were infected with *H. pylori*. There was no association between infection with *H. pylori* and existence of hiatal hernia in EAC cases.

## Conclusion:

Gender, age, gastritis, and *H. Pylori* infection were not significantly associated with symptomatic or endoscopic GERD. None of the demographic or clinical findings were significantly associated with BE. Male gender was more frequent in both EAC and SCC cases. Patients with SCC were significantly older and had lower risk of hiatal hernia. *H. pylori* infection was a risk factor of pathologic findings consistent with gastritis. We believe there are still very few studies about Barrett, GERD, EAC and SCC prevalence and risk factors in AAs. Our study shed a little more light on these topics but larger size studies are needed in African Americans.

## Supplementary Material

1

## Figures and Tables

**Table 1:** Demographic, clinical and pathologic findings in the study cohort

Male, no (%)	610 (49%)
Over 60 years old, no (%)	641 (51%)
Age, median (IQR) years	61 (52–70)
GERD clinical symptoms, no (%)	516 (41%)
Hiatal hernia, no (%)	278 (22%)
*Helicobacter pylori*, no (%)	224 (23%) *
Endoscopic Esophagitis, no (%)	430 (34%)
Endoscopic gastritis, no (%)	874 (63%)
Barrett, no (%)	8 (0.6%)
Adenocarcinoma, no (%)	13 (1.0%)
Squamous cell carcinoma, no (%)	36 (2.9%)
Pathologic gastritis, no (%)	448 (42%) **

* n=993

** n=1079

## References

[1] Todd JA, de Caestecker J, Jankowski J (2003) Gastro-esophageal reflux disease and bile acids. J Pediatr Gastroenterol Nutr 36: 172–174. 12548050 10.1097/00005176-200302000-00004

[2] Yung E, Li X, Chandrasoma P (2021) Intestinal Metaplasia of the “Cardia”: Accurate Differentiation of Gastric or Esophageal Origin With an Expanded Biopsy Protocol. Am J Surg Pathol 45: 945–950. 33739789 10.1097/PAS.0000000000001665

[3] Clarrett DM, Hachem C (2018) Gastroesophageal Reflux Disease (GERD). Mo Med 115: 214–218. 30228725 PMC6140167

[4] Greenspan NR (2011) Gastroesophageal reflux disease: endoscopy, duration of treatment, and choice of PPI. Med Health R I 94: 92–94. 21667598

[5] Avidan B, Sonnenberg A, Schnell TG, Chejfec G, Metz A, (2002) Hiatal hernia size, Barrett’s length, and severity of acid reflux are all risk factors for esophageal adenocarcinoma. Am J Gastroenterol 97: 1930–1936. 12190156 10.1111/j.1572-0241.2002.05902.x

[6] Harada K, Rogers JE, Iwatsuki M, Yamashita K, Baba H, (2020) Recent advances in treating oesophageal cancer. F1000Res 9. 10.12688/f1000research.22926.1 PMC7531047 33042518

[7] Patel A, Gyawali CP (2019) Screening for Barrett’s Esophagus: Balancing Clinical Value and Cost-effectiveness. J Neurogastroenterol Motil 25: 181–188. 30827080 10.5056/jnm18156 PMC6474698

[8] Sawaya RA, Macgill A, Parkman HP, Friedenberg FK (2012) Use of the Montreal global definition as an assessment of quality of life in reflux disease. Dis Esophagus 25: 477–483. 21966890 10.1111/j.1442-2050.2011.01271.x PMC3252470

[9] Ford AC, Forman D, Reynolds PD, Cooper BT, Moayyedi P (2005) Ethnicity, gender, and socioeconomic status as risk factors for esophagitis and Barrett’s esophagus. Am J Epidemiol 162: 454–460. 16076833 10.1093/aje/kwi218

[10] Naini BV, Souza RF, Odze RD (2016) Barrett’s Esophagus: A Comprehensive and Contemporary Review for Pathologists. Am J Surg Pathol 40: e45–66. 26813745 10.1097/PAS.0000000000000598 PMC4833583

[11] Guardino JM, Khandwala F, Lopez R, Wachsberger DM, Richter JE, (2006) Barrett’s esophagus at a tertiary care center: association of age on incidence and prevalence of dysplasia and adenocarcinoma. Am J Gastroenterol 101: 2187–2193. 17032182 10.1111/j.1572-0241.2006.00736.x

[12] Deurloo JA, Ekkelkamp S, Bartelsman JF, Ten Kate FJ, Schoorl M, (2003) Gastroesophageal reflux: prevalence in adults older than 28 years after correction of esophageal atresia. Ann Surg 238: 686–689. 14578730 10.1097/01.sla.0000094303.07910.05 PMC1356146

[13] Nguyen TH, Thrift AP, Ramsey D, Green L, Shaib YH, (2014) Risk factors for Barrett’s esophagus compared between African Americans and non-Hispanic Whites. Am J Gastroenterol 109: 1870–1880. 25420546 10.1038/ajg.2014.351

[14] Ferrer-Torres D, Nancarrow DJ, Steinberg H, Wang Z, Kuick R, (2019) Constitutively Higher Level of GSTT2 in Esophageal Tissues From African Americans Protects Cells Against DNA Damage. Gastroenterology 156: 1404–1415. 30578782 10.1053/j.gastro.2018.12.004 PMC6441633

[15] Bergholt MS, Zheng W, Ho KY, Teh M, Yeoh KG, (2014) Fiberoptic confocal raman spectroscopy for real-time in vivo diagnosis of dysplasia in Barrett’s esophagus. Gastroenterology 146: 27–32. 24216327 10.1053/j.gastro.2013.11.002

[16] Jung KW, Talley NJ, Romero Y, Katzka DA, Schleck CD, (2011) Epidemiology and natural history of intestinal metaplasia of the gastroesophageal junction and Barrett’s esophagus: a population-based study. Am J Gastroenterol 106: 1447–1455. 21483461 10.1038/ajg.2011.130 PMC3150349

[17] Singh T, Sanghi V, Thota PN (2019) Current management of Barrett esophagus and esophageal adenocarcinoma. Cleve Clin J Med 86: 724–732. 31710585 10.3949/ccjm.86a.18106 PMC7050471

[18] Shaheen NJ, Falk GW, Iyer PG, Souza RF, Yadlapati RH, (2022) Diagnosis and Management of Barrett’s Esophagus: An Updated ACG Guideline. Am J Gastroenterol 117: 559–587. 35354777 10.14309/ajg.0000000000001680 PMC10259184

[19] Steele D, Baig KKK, Peter S (2019) Evolving screening and surveillance techniques for Barrett’s esophagus. World J Gastroenterol 25: 2045–2057. 31114132 10.3748/wjg.v25.i17.2045 PMC6506582

[20] Thota PN, Zackria S, Sanaka MR, Patil D, Goldblum J, (2017) Racial Disparity in the Sex Distribution, the Prevalence, and the Incidence of Dysplasia in Barrett’s Esophagus. J Clin Gastroenterol 51: 402–406. 27306940 10.1097/MCG.0000000000000559 PMC5159321

[21] Qumseya BJ, Bukannan A, Gendy S, Ahemd Y, Sultan S, (2019) Systematic review and meta-analysis of prevalence and risk factors for Barrett’s esophagus. Gastrointest Endosc 90: 707–717 e701. 31152737 10.1016/j.gie.2019.05.030

[22] Inadomi JM, Saxena N (2018) Screening and Surveillance for Barrett’s Esophagus: Is It Cost-Effective? Dig Dis Sci 63: 2094–2104. 29948571 10.1007/s10620-018-5148-7

[23] Mohammed I, Nightingale P, Trudgill NJ (2005) Risk factors for gastro-oesophageal reflux disease symptoms: a community study. Aliment Pharmacol Ther 21: 821–827. 15801917 10.1111/j.1365-2036.2005.02426.x

[24] Rodriguez S, Mattek N, Lieberman D, Fennerty B, Eisen G (2008) Barrett’s esophagus on repeat endoscopy: should we look more than once? Am J Gastroenterol 103: 1892–1897. 18564120 10.1111/j.1572-0241.2008.01892.x PMC3922226

[25] Wang YR, Loftus EV Jr, Judge TA, Peikin SR (2016) Rate and Predictors of Interval Esophageal and Gastric Cancers after Esophagogastroduodenoscopy in the United States. Digestion 94: 176–180. 27871069 10.1159/000452794

[26] Jemal A, Bray F, Center MM, Ferlay J, Ward E, (2011) Global cancer statistics. CA Cancer J Clin 61: 69–90. 21296855 10.3322/caac.20107

[27] Cook MB, Chow WH, Devesa SS (2009) Oesophageal cancer incidence in the United States by race, sex, and histologic type, 1977–2005. Br J Cancer 101: 855–859. 19672254 10.1038/sj.bjc.6605246 PMC2736840

[28] Thrift AP, Whiteman DC (2012) The incidence of esophageal adenocarcinoma continues to rise: analysis of period and birth cohort effects on recent trends. Ann Oncol 23: 3155–3162. 22847812 10.1093/annonc/mds181

[29] Kendall BJ, Whiteman DC (2006) Temporal changes in the endoscopic frequency of new cases of Barrett’s esophagus in an Australian health region. Am J Gastroenterol 101: 1178–1182. 16771933 10.1111/j.1572-0241.2006.00548.x

[30] Alkaddour A, Palacio C, Vega KJ (2018) Risk of histologic Barrett’s esophagus between African Americans and non-Hispanic whites: A meta-analysis. United European Gastroenterol J 6: 22–28. 10.1177/2050640617707862 PMC5802672 29435310

[31] Chang CS, Poon SK, Lien HC, Chen GH (1997) The incidence of reflux esophagitis among the Chinese. Am J Gastroenterol 92: 668–671. 9128320

[32] Yeh C, Hsu CT, Ho AS, Sampliner RE, Fass R (1997) Erosive esophagitis and Barrett’s esophagus in Taiwan: a higher frequency than expected. Dig Dis Sci 42: 702–706. 9125635 10.1023/a:1018835324210

[33] Ho KY, Kang JY, Seow A (1998) Prevalence of gastrointestinal symptoms in a multiracial Asian population, with particular reference to reflux-type symptoms. Am J Gastroenterol 93: 1816–1822. 9772037 10.1111/j.1572-0241.1998.00526.x

[34] Rajendra S, Kutty K, Karim N (2004) Ethnic differences in the prevalence of endoscopic esophagitis and Barrett’s esophagus: the long and short of it all. Dig Dis Sci 49: 237–242. 15104363 10.1023/b:ddas.0000017444.30792.94

[35] Rosaida MS, Goh KL (2004) Gastro-oesophageal reflux disease, reflux oesophagitis and non-erosive reflux disease in a multiracial Asian population: a prospective, endoscopy based study. Eur J Gastroenterol Hepatol 16: 495–501. 15097043 10.1097/00042737-200405000-00010

[36] de Bortoli N, Tolone S, Frazzoni M, Martinucci I, Sgherri G, (2018) Gastroesophageal reflux disease, functional dyspepsia and irritable bowel syndrome: common overlapping gastrointestinal disorders. Ann Gastroenterol 31: 639–648. 30386113 10.20524/aog.2018.0314 PMC6191868

[37] de Bortoli N, Natali V, Melissari S, Simonetti N, Tapete G, (2017) Overlap of GERD and gastrointestinal functional disorders. Minerva Gastroenterol Dietol 63: 205–220. 28260354 10.23736/S1121-421X.17.02398-4

[38] Corona E, Yang L, Esrailian E, Ghassemi KA, Conklin JL, (2021) Trends in Esophageal Cancer Mortality and Stage at Diagnosis by Race and Ethnicity in the United States. Cancer Causes Control 32: 883–894. 34003396 10.1007/s10552-021-01443-z PMC8236464

[39] Dulai GS, Guha S, Kahn KL, Gornbein J, Weinstein WM (2002) Preoperative prevalence of Barrett’s esophagus in esophageal adenocarcinoma: a systematic review. Gastroenterology 122: 26–33. 11781277 10.1053/gast.2002.30297

[40] Brim H, Zahaf M, Laiyemo AO, Nouraie M, Perez-Perez GI, (2014) Gastric Helicobacter pylori infection associates with an increased risk of colorectal polyps in African Americans. BMC Cancer 14: 296. 24774100 10.1186/1471-2407-14-296 PMC4022546

[41] Khoury JE, Chisholm S, Jamal MM, Palacio C, Pudhota S, (2012) African Americans with Barrett’s esophagus are less likely to have dysplasia at biopsy. Dig Dis Sci 57: 419–423. 21909989 10.1007/s10620-011-1900-y

[42] Abrams JA, Fields S, Lightdale CJ, Neugut AI (2008) Racial and ethnic disparities in the prevalence of Barrett’s esophagus among patients who undergo upper endoscopy. Clin Gastroenterol Hepatol 6: 30–34. 18063419 10.1016/j.cgh.2007.10.006 PMC3712273

[43] Jones B, Williams JL, Komanduri S, Muthusamy VR, Shaheen NJ, (2021) Racial Disparities in Adherence to Quality Indicators in Barrett’s Esophagus: An Analysis Using the GIQuIC National Benchmarking Registry. Am J Gastroenterol 116: 1201–1210. 33767105 10.14309/ajg.0000000000001230

[44] Wu X, Chen VW, Andrews PA, Ruiz B, Correa P (2007) Incidence of esophageal and gastric cancers among Hispanics, non-Hispanic whites and non-Hispanic blacks in the United States: subsite and histology differences. Cancer Causes Control 18: 585–593. 17406989 10.1007/s10552-007-9000-1

