# Supplementary material for: Exploring the Incidence and Associated Risk Factors of Barrett’s Esophagus in African Americans: A Retrospective Study
Source: Rep Glob Health Res. Author manuscript; Available in PMC 2023 Dec 2. (PMC10691964; doi:10.29011/2690-9480.100164)
Supplement: 1 [file NIHMS1930135-supplement-1.pdf]

Supplementary Table1A-1D: Demographic and clinico-pathologic features correlations with absence (negative - sign) or presence (positive + sign) of GERD symptoms (-, N=737), GERD symptoms (+, N=516); Endoscopic Esophagitis (–, N=823), Endoscopic Esophagitis (+, N=430); Barrett (–, N=1,245), Barrett (+, N=8); Adenocarcinoma (-, N=1,240), Adenocarcinoma (+, N=13); SCC (–, N=1217), SCC (+, N=36); Pathologic gastritis (–, N=631), Pathologic gastritis (+,N=448).

**Supplementary Table 1A: Demographic and clinico-pathologic findings correlations with GERD clinical symptoms (a) and Endoscopic Esophagitis (b)**			
**a)**	**GERD symptoms – (N=737)**	**GERD symptoms + (N=516)**	**P value**
Male, no (%)	368 (50%)	242 (47%)	0.3
Over 60 years old, no (%)	388 (53%)	253 (49%)	0.2
Hiatal hernia, no (%)	156 (21%)	122 (24%)	0.3
Helicobacter pylori, no (%)	123 (21%)1	101 (26%)[2]	0.07
Pathologic gastritis, no (%)	261 (41%)3	187 (43%)[4]	0.5
**b)**	**Endoscopic Esophagitis –(N=823)**	**Endoscopic Esophagitis +(N=430)**	**P value**
Male, no (%)	404 (49%)	206 (48%)	0.7
Over 60 years old, no (%)	419 (51%)	222 (52%)	0.8
Hiatal hernia, no (%)	164 (20%)	114 (27%)	0.008
Helicobacter pylori, no (%)	160 (24%)5	64 (19%)6	0.09
Pathologic gastritis, no (%)	301 (42%)7	147 (40%)8	0.6
GERD symptoms, no (%)	343 (42%)	173 (40%)	0.6
[1] n= 597 [2] n= 396 [3] n=641 [4] n=438			
[5] n= 662 [6] n= 331 [7] n=714 [8] n=365			
**Supplementary Table 1B: Association between demographic, clinico-pathologic findings, and Barrett**			
	Barrett –(N=1,245)	Barrett +(N=8)	P value
Male, no (%)	606 (49%)	4 (50%)	0.9
Over 60 years old, no (%)	635 (51%)	6 (75%)	0.3
Hiatal hernia, no (%)	275 (22%)	3 (38%)	0.4
Helicobacter pylori, no (%)	224 (23%)[1]	0 [2]	0.4
Pathologic gastritis, no (%)	446 (42%)[3]	2 (25%)	0.5
GERD symptoms, no (%)	513 (41%)	3 (38%)	0.9
[1] n= 986 [2] n= 7 [3] n=1071			
**Supplementary Table 1C: Demographic and clinico-pathologic findings correlations with esophagus adenocarcinoma (a) and esophagus squamous carcinoma (b)**			
**a)**	**Adenocarcinoma- (N=1,240)**	**Adenocarcinoma+(N=13)**	**P value**
Male, no (%) B35:B36	600 (48%)	10 (77%)	0.05
Over 60 years old, no (%)	632 (51%)	9 (69%)	0.3
Hiatal hernia, no (%)	278 (22%)	0	0.09
Helicobacter pylori, no (%)	222 (23%)[1]	2 (22%)[2]	0.9

Pathologic gastritis, no (%)	445 (42%)[3]	3 (27%)[4]	0.4
**b)**	**SCC –(N=1217)**	**SCC +(N=36)**	**P value**
Male, no (%)	583 (48%)	27 (75%)	0.002
Over 60 years old, no (%)	609 (50%)	32 (89%)	<0.001
Hiatal hernia, no (%)	275 (23%)	3 (8%)	0.042
Helicobacter pylori, no (%)	213 (22%)[5]	11 (35%)[6]	0.08
Pathologic gastritis, no (%)	434 (41%)[7]	14 (42%)[8]	0.9
[1] n= 984 [2] n= 9 [3] n=1068 [4] n=11			
[5] n= 962 [6] n= 31 [7] n=1046 [8] n=33			
**Supplementary Table 1D: Association between demographic, clinico-pathologic findings, and gastritis**			
	**Pathologic gastritis –(N=631)**	**Pathologic gastritis +(N=448)**	**P value**
Male, no (%)	323 (51%)	211 (47%)	0.2
Over 60 years old, no (%)	329 (52%)	228 (51%)	0.7
Hiatal hernia, no (%)	144 (23%)	94 (21%)	0.5
Helicobacter pylori, no (%)	3 (0.5%)	220 (58%)	<0.001
Endoscopic gastritis, no (%)	438 (69%)	312 (70%)	0.9

Discussion:

In this hospital-based 11 years study of 1,253 patients' clinical and pathology reports, more female patients sought EGD screening than males, probably reflecting a better health awareness in females and more resilience in male AA patients as it relates to upper GI discomfort. Additionally, it was found that BE was more likely in males than in females based on histologic evaluation (2.9% vs. 0.8%, P = 0.0001) [41]. Patients undergoing EGD without any symptoms represented more than half of the study cohort (59%). Hiatal hernia, infection with *H. pylori*, endoscopic and pathologic gastritis were found in these 737 symptom-free patients which likely reflect that in the general AA population, many of these findings are underestimated.

In our study, the prevalence of endoscopic 874 (63%), pathologic 448 (42%), gastritis, hiatal hernia 278 (22%) and infection with *H. pylori* 224 (23%) were high, while BE 8 (0.6%), EAC 13 (1.0%) and SCC 36 (2.9%) prevalence were low compared to Non-Hispanic Whites [13]. Patients with GERD symptoms (516) showed more infection with *H. pylori* 101(26%) and pathological gastritis 187 (43%) when compared to NHWs. Although 737 patients had no GERD associated, there were significant rates of *H. pylori* infection 156 (21%) and gastritis on biopsy 261(41%). The prevalence of BE in African Americans has been addressed only in 2 prior published studies [13,42]. In a cross-sectional EGD study conducted at the Michael E. DeBakey Veteran Affairs Medical Center (MEDVAMC) in Houston, Texas, (301 BE cases and 1,651 controls). NHWs displayed a significantly higher BE prevalence than AAs (21.3 vs. 5.0%; P <0.001). Jones et al., 2021 have reported that based on histologically confirmed samples, blacks (n=3,957) had shorter BE lengths (1.61 vs 2.35 cm, P < 0.01) and were less likely with dysplasia than whites (n=96,891) [43].

In our study, there were 8 BE cases in 1,253 patients. From these 8 BE cases, the gender distribution was equal but none of them had infection with *H. pylori*. Ashktorab et al. previously reported that in AAs, *H. pylori* infection was found to be a protective factor for reflux esophagitis which is a precursor for BE. In concordance with our previous report, other recent report demonstrated that the protective factor of BE was differential expression of glutathione S-transferase theta 2 (GSTT2) gene in African Americans compared with European Americans [14].

More than half of cases with BE were older than 60 and 3 (38%) had hiatal hernia. Age over 50 years and hiatal hernia are risk factors for BE both in NHWs and AAs. NHWs were more likely to be male, and less likely to have *H. pylori* infection (P <0.001) [42]. NHWs were more prone to have long-segment BE and dysplasia than AAs. Independent BE risk factors for AAs, hiatus hernia ≥3 cm (OR 4.12; 95% CI, 1.57–10.81) and H. pylori (OR, 0.64; 95% CI, 0.41–0.99) were statistically significantly associated with BE risk for NHWs. Among all cases and controls, race was a risk factor for BE, independent of other BE risk factors (OR for AAs, 0.26; 95%).
